# People Who Self-Reported Testing HIV-Positive but Tested HIV-Negative: A Multi-Country Puzzle of Data, Serology, and Ethics, 2015–2021

**DOI:** 10.3390/tropicalmed9090220

**Published:** 2024-09-19

**Authors:** Melissa Metz, Vivian Hope Among, Tafadzwa Dzinamarira, Faith Ussery, Peter Nkurunziza, Janet Bahizi, Samuel Biraro, Francis M. Ogollah, Joshua Musinguzi, Wilford Kirungi, Mary Naluguza, Christina Mwangi, Sehin Birhanu, Lisa J. Nelson, Herbert Longwe, Frieda Sara Winterhalter, Andrew C. Voetsch, Bharat S. Parekh, Hetal K. Patel, Yen T. Duong, Rachel Bray, Shannon M. Farley

**Affiliations:** 1 ICAP at Columbia University, New York, NY 10032, USA; sara.winterhalter@gmail.com (F.S.W.); smf2026@cumc.columbia.edu (S.M.F.); 2 Heart to Heart International, Lenexa, KS 66219, USA; hopevivians2@gmail.com; 3 ICAP Rwanda, Kigali, Rwanda; td2581@cumc.columbia.edu; 4 Division of Global HIV and TB, Global Health Center, US Centers for Disease Control and Prevention (CDC), Atlanta, GA 30329, USA; inh3@cdc.gov (F.U.); vjw2@cdc.gov (S.B.); aav6@cdc.gov (A.C.V.); bsp1@cdc.gov (B.S.P.); byg7@cdc.gov (H.K.P.); 5 ICAP Uganda, Plot 1 Lourdel Rd, 5th Floor Lourdel Towers, Kampala, Uganda; peter.nkurunziza@gmail.com (P.N.); janetkbahizi@gmail.com (J.B.); samuel.biraro@gmail.com (S.B.); 6 ICAP Malawi, Lilongwe P.O. Box 31604, Malawi; fo2201@cumc.columbia.edu; 7 Uganda Ministry of Health, Kampala P.O. Box 7272, Ugandawkirungi@starcom.co.ug (W.K.); 8 Division of Global HIV and TB, Global Health Center, US Centers for Disease Control and Prevention (CDC), Kampala P.O. Box 7007, Uganda; yrs0@cdc.gov (M.N.); lbn9@cdc.gov (L.J.N.); 9 ICAP South Africa, Erasmuskloof, Pretoria P.O Box 11203, South Africa; hl2954@cumc.columbia.edu

**Keywords:** HIV, population-based surveys, PHIA, HIV rapid tests, self-report status, Uganda, algorithms, diagnostic markers, misdiagnosis

## Abstract

During population-based HIV impact assessments (PHIAs), some participants who self-reported testing HIV-positive (PSRP) tested negative in one or more subsequent survey HIV tests. These unexpected discrepancies between their self-reported results and the survey results draw into question the validity of either the self-reported status or the test results. We analyzed PSRP with negative test results aged 15–59 years old using data collected from 2015 to 2021 in 13 countries, assessing prevalence, self-report status, survey HIV status, viral load, rapid tests and confirmatory tests, and answers to follow-up questions (such as years on treatment). Across these surveys, 19,026 participants were PSRP, and 256 (1.3%) of these were concluded to be HIV-negative after additional survey-based testing and review. PSRP determined to be HIV-negative trended higher in countries with a higher HIV prevalence, but their number was small enough that accepting self-reported HIV-positive status without testing would not have significantly affected the prevalence estimates for HIV or viral load suppression. Additionally, using more detailed information for Uganda, we examined 107 PSRP with any negative test results and found no significant correlation with years on treatment or age. Using these details, we examined support for the possible reasons for these discrepancies beyond misdiagnosis and false reporting. These findings suggest that those conducting surveys would benefit from a nuanced understanding of HIV testing among PSRP to conduct surveys ethically and produce high-quality results.

## 1. Introduction

Surveys designed to measure HIV seroprevalence using HIV tests generally include questions regarding self-reported HIV testing history, for example, to determine awareness. There is the potential for discrepancies between a survey’s HIV test results and self-reported HIV testing history. This can occur for several reasons. Cases in which an individual self-reports as previously testing HIV-negative but tests HIV-positive can be the result of a new HIV diagnosis but can also be the result of falsely reporting a negative HIV status [1]. Neither of these causes is surprising. Conversely, it is possible for individuals to self-report that they have previously tested HIV-positive while testing suggests that they are negative [2]; participants who self-report as positive (PSRP) but test negative are more perplexing and less frequently studied, and this contradiction can often be resolved with additional testing to detect antiretrovirals (ARVs) [1] or a low viral load (VL) or by obtaining access to and reviewing previous clinical records [3]. These cases also trigger ethical considerations of how to present conflicting information to a participant about their clinical status in a way that will not cause harm.

Studies that assess the accuracy of HIV self-report status rarely report on PSRP who test negative and generally do not include retesting PSRP discrepancies with a variety of tests. A 2023 paper linked 1657 participants from a 2018 population-based survey in South Africa with clinical data from local healthcare facilities, focusing on self-reporting of recent HIV testing. Among 250 PSRP, 3 (1.2%) had past clinical records showing they previously tested negative for HIV; these were attributed to respondents’ misunderstanding of the questions or data entry errors [3]. A 2014 report on the validity of self-reported HIV status in Malawi and Uganda among 33,105 respondents found substantial (24–40%) underreporting of HIV-positive status but did not examine those who tested negative [1]. A study in rural South Africa reviewed 5059 records from 2014 to 2015 and found that 94.1% of PSRP tested positive on screening and confirmatory tests but did not examine the 5.9% of PSRP who tested HIV-negative [2]. A study in rural northern Malawi reviewing 17,856 records from 2007 to 2018 reported that 98.0% of PSRP had records of previous HIV-positive rapid tests (RTs) and suggested that the remaining 2.0% had seroconverted after these previous tests; they did not retest those individuals [4]. A 2018 study in rural Mozambique examined those who did not disclose their HIV-positive status before repeat testing and did not examine PSRP [5]. Notably, none of these studies retested or further investigated the PSRP with negative test results.

Conversely, two previous papers leveraging data from population-based HIV impact assessment (PHIA) surveys examined PSRP with discrepancies. A 2022 paper reviewing the accuracy of self-reported HIV status in the 2018 Nigeria HIV/AIDS Indicator and Impact Survey found that 64 PSRP who tested HIV-negative in RTs also tested negative in polymerase chain reaction (PCR) tests [6]. Similarly, a 2024 paper reviewing 16,630 survey participants across 11 countries who self-reported as HIV-positive examined 198 (1.4%) who tested HIV-negative, finding that they were younger and less likely to have received ARVs [7].

Using data from 15 PHIA surveys in 13 countries, this analysis focuses on survey participants from 2015 to 2021 who reported that they had previously tested HIV-positive while the survey tests yielded negative or inconclusive (non-positive) results for them. This two-phased analysis first examined the magnitude of the problem across these 15 surveys and then examined the participants from one country, Uganda, in more detail. Determining true HIV status with these self-report discrepancies is important for ethically supporting participants’ clinical care, for improving survey data quality and completeness, and for understanding how HIV infection presents at later stages and/or after longer times on treatment better.

## 2. Materials and Methods

**Figure 1:**
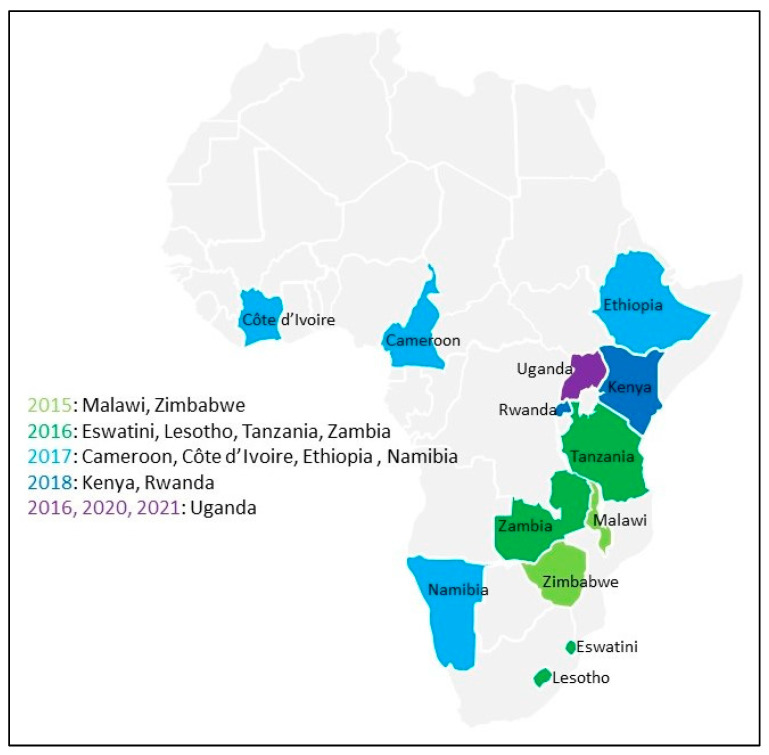
Thirteen countries with public data for population-based HIV impact assessments; 2015: Malawi and Zimbabwe; 2016: Eswatini, Lesotho, Tanzania, and Zambia; 2017: Cameroon, Côte d’Ivoire, Ethiopia, and Namibia; 2018: Kenya and Rwanda; 2016, 2020, and 2021: Uganda, for which pre-published data from the second survey and the refugee survey are used.

The PHIAs [8] assessed the status of the HIV epidemic—including its prevalence, incidence, and population viral load suppression—and the impact of public health programs in several countries. The surveys were cross-sectional and household-based and used a multi-stage cluster sampling design, described in detail elsewhere [9,10,11,12,13,14,15,16,17,18,19]. After obtaining informed consent, the interviewers collected demographic, behavioral, and clinical information from eligible household participants, and blood samples were collected for HIV testing. The data for the first 13 surveys (2015: Malawi and Zimbabwe; 2016: Eswatini, Lesotho, Tanzania, Uganda, and Zambia; 2017: Cameroon, Côte d’Ivoire, Ethiopia, and Namibia; 2018: Kenya and Rwanda) (Figure 1) have been published [20].

This paper leverages these public datasets and examines data from the second Ugandan PHIA (UPHIA 2020) and a survey of refugees in Uganda (RUPHIA 2021) more closely, though these datasets are not yet public; they were used with the approval of the principal investigators. The three Ugandan surveys provided a unique set of comparable process data available to the investigator institutions. Such data included results from all the survey tests which were used to determine HIV status for the final analysis and the public datasets: HIV RTs; repeat RTs for quality assurance (QA) and discrepancy resolution; Geenius (Geenius HIV 1/2 Supplemental Assay; Bio-Rad, Hercules, CA, USA) confirmatory testing; total nucleic acid polymerase chain reaction (TNA PCR); and (unique among these countries) Western Blot (WB), as well as VL and CD4 testing and responses to the ARV questions participants who had self-reported previously testing HIV-positive were asked.

Multiple questions were asked to determine whether a participant was aware of their HIV-positive status and how long they had known about it. Table 1 shows the specific questions asked in the Ugandan surveys as an example; the surveys in the other countries generally used the same questions. For Uganda in 2020 and Uganda Refugees in 2021 (and other surveys not included in this analysis), an additional question was added to identify those who may have been diagnosed so long ago that they did not explicitly remember being tested (“Has a health care provider ever told you that you have HIV?”). If the answer to any of these questions was “yes” or a positive response, the skip logic in the questionnaire triggered questions such as whether the participant had ever or was currently taking ARVs, and if so, when they started taking ARVs.

Across all the PHIAs, blood was collected for home-based HIV rapid testing (details below) and counseling, with the results immediately returned to the participants. Blood specimens that tested positive or inconclusive for HIV underwent Geenius testing at satellite laboratories and additional tests such as HIV VL tests at a national reference laboratory or a similar laboratory. A subset of specimens, including all that were inconclusive for HIV, were retested at satellite laboratories using the same HIV RT algorithm used in the households. Blood specimens that were positive were tested for ARVs; for Uganda 2020 and Uganda Refugees 2021, all the PSRPs’ blood samples were tested for ARVs, including those concluded to be negative after the discrepancy resolution process. Additional details on the PHIA testing and discrepancy resolution processes are available elsewhere [9,15,21,22].

**Figure 2:**
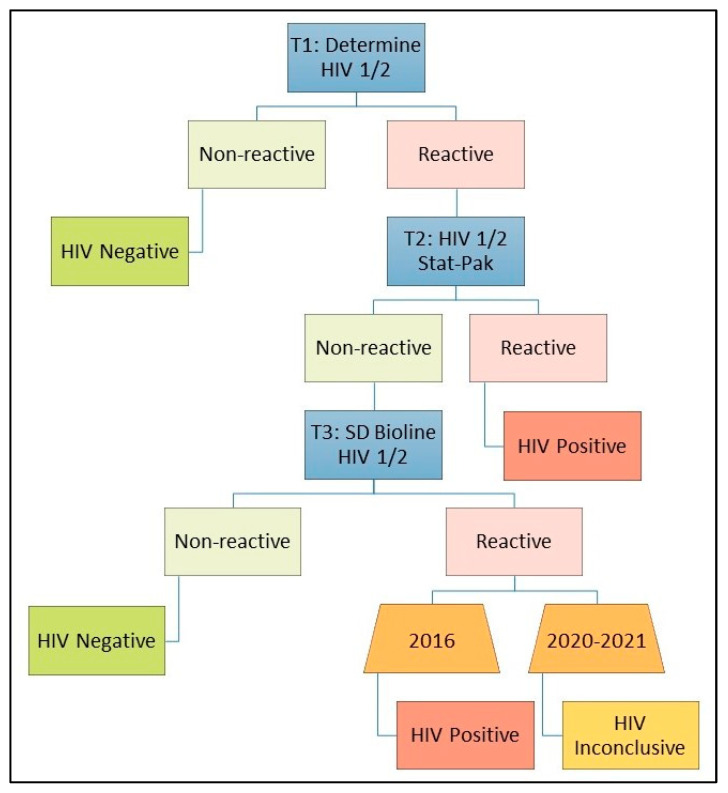
National HIV testing algorithm in Uganda, as used for population-based HIV impact assessment surveys in 2016, 2020, and 2021.

HIV RTs were used according to each country’s national algorithm at the time of the data collection. These algorithms, detailed elsewhere [22,23], differ by country based on country-specific needs, for example, tests that perform better in a high-prevalence country or based on test availability and affordability; additionally, national HIV algorithms can change over time. For example, from 2010 to 2015 in Uganda, a thorough evaluation was carried out of the existing national HIV RT algorithm and the specific tests being used, comparing these to well-performing and cost-effective alternatives; this led to a new national algorithm [24], which was used during the 2016 PHIA survey. Another change was made before Uganda’s 2020 PHIA and 2021 Refugees PHIA; the same tests were used for all three Ugandan surveys: Determine HIV-1/2 (Abbott Molecular Inc., Des Plaines, IL, USA) as the screening test (T1), HIV 1/2 Stat-Pak (Chembio Diagnostic Systems, Medford, New York, NY, USA) for confirmation (T2), and SD Bioline HIV-1/2 3.0 (Standard Diagnostics, Inc., Kyonggido, Republic of Korea) in case of discordance (T3) (Figure 2). In all three, the same conclusion was drawn for T1 non-reactive (HIV-negative); T1 and T2 reactive (positive); and T1 reactive, T2 non-reactive, and T3 non-reactive (negative) results. However, the conclusion for T1 reactive, T2 non-reactive, and T3 reactive changed; in 2016, the conclusion was HIV-positive, but in the 2020 and 2021 surveys, for T3 reactive results, the algorithm indicated these were HIV-inconclusive (Figure 2). This was based on the World Health Organization (WHO) guidelines and recommendations for the testing strategy/algorithm for HIV diagnosis in high- and low-prevalence settings [25].

For the PHIA surveys, all the participants with an inconclusive result in the household testing were referred to a health facility of their choice for repeat testing and counseling after about 14 days per the national guidelines and as an ethical obligation to support diagnosis of these participants potentially living with HIV. For survey purposes, samples with inconclusive test results were retested as part of the discrepancy resolution process described below.

The PHIA interviewers and counselors were trained in ethical methods for handling cases where a participant self-reported a previous positive test but their household test results were unexpectedly negative or inconclusive. The participant was not told that they had tested negative or inconclusively at their household, as this might have caused harm if they suddenly stopped treatment. Instead, they were told that the survey team would conduct additional testing in the laboratory and revisit the household with more information; the survey team concluded it was ethically important to share additional information which could improve the participant’s clinical care, whether to reinforce their HIV diagnosis or to question it.

In order to understand the cases where any survey tests for the PSRP were discrepant, we used a truth table to ensure we considered all possibilities (Table 2).

There are two scenarios if the self-report was true and the test was correct; both involve inaccurate data entry. Recorded survey responses have a small chance of being inaccurate, even when using experienced staff, well-programmed questionnaires, and comprehensive training and monitoring [15]; inaccurate responses can be recorded due to misunderstandings or transcription errors. In scenario 1, the tests produced true positive results but were read incorrectly or inadvertently entered as negative during the data collection; this scenario can include sample mix-ups (the results being entered for the wrong participant). In scenario 2, the participant reported no previous positive tests, but their response was recorded incorrectly during the data collection.

The next two scenarios (self-report is false, test is correct) involved true negative survey results but the positive self-report being false. In scenario 3, the initial diagnosis was falsely positive. In scenario 4, the participant misstated their previous test results.

In the final scenario (self-report is true, test is incorrect), the positive self-report was true, but the negative test result was false, i.e., one or more of the tests failed to detect the HIV infection.

The discrepancy resolution process, by running additional tests and reviewing the results closely, attempted to distinguish between these scenarios. Addressing self-report discrepancies was part of a larger process of QA [21] and discrepancy resolution. Discrepancies were reviewed by a discrepancy resolution team, which included senior PHIA laboratory advisors, the survey-specific laboratory advisor, and members of the data team.

**Figure 3:**
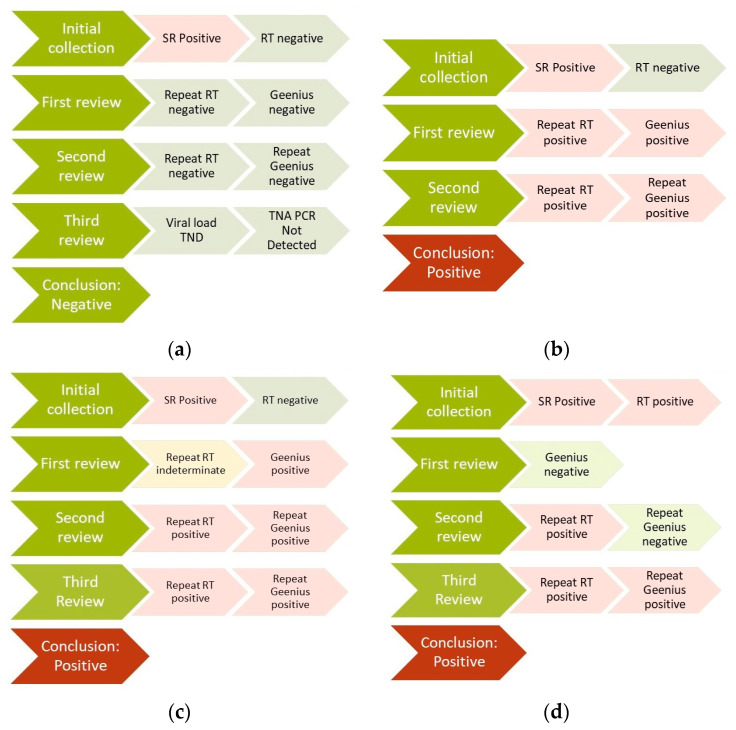
Examples of retesting scenarios for self-report discrepancies during population-based HIV impact assessments in multiple countries: (**a**) all tests negative; (**b**) possible household testing or recording errors; (**c**) inconsistent rapid test results; (**d**) inconsistent Geenius results. RT: rapid test; SR: self-report; TNA PCR: total nucleic acid polymerase chain reaction; TND: Target Not Detected.

The discrepancy resolution team developed a detailed but flexible process to make a conclusion regarding HIV status both for the participant and for the survey, which included repeating the HIV RT algorithm, performing and repeating Geenius testing, performing TNA PCR tests, and using quantitative VL testing. Exponentially, these 4 tests could produce up to 64 combinations of positive versus negative, many of which have been seen across PHIA surveys; Figure 3 shows four common examples. For Uganda in 2020, WB testing was also run, e.g., for PSRP with inconclusive RTs or Geenius tests since there were a larger number than expected. Once sufficient data were available, the team decided on each result to use for the survey, as well as whether to revisit the participant; for example, for (a) (all laboratory-based tests being negative), negative PCR results would be returned, and such participants were counseled that any changes in treatment based on these results should be made under the care of a medical professional at a health facility to reduce the risk of harm.

### Analyses

We quantified the number of PSRP, the number who were concluded to be positive for HIV, and the number who were virally suppressed aged 15–59 years old across all 15 surveys, for each survey and overall. We calculated the percent of PSRP concluded to be negative as a proportion of all the participants and as a proportion of all of the PSRP, for each survey and overall.

Two sensitivity analyses were conducted for this same population, the first for prevalence of HIV. We used the SAS 9.4 (SAS Institute, Cary, NC, USA) procedure surveymeans with weighting twice, first to measure the survey HIV prevalence using the final survey HIV status and secondly using the conditional assumption that all the PSRP were living with HIV, i.e., adding the PSRP who were concluded to be negative to all the participants who were concluded to be HIV-positive.

The trendline for the percent of participants who were PSRP concluded to be negative by survey HIV prevalence was calculated using Excel version 2408 (Microsoft, Redmond, WA, USA).

The second sensitivity analysis examined the prevalence of HIV VL suppression (VLS), defined as a VL <1000 copies/milliliter. In later PHIA surveys, VL testing was more consistently requested for self-report discrepancies; among those concluded to be negative, the available VL values were generally “Target Not Detected” (TND) or below the lower limit of detection of the instrument. If they had been concluded to be positive, these PSRP thus would have been considered virally suppressed. Again, we used the SAS procedure surveymeans with weighting twice, first to measure the survey VLS prevalence using the final survey VLS status and then again using the conditional assumption that all the PSRP were HIV-positive and virally suppressed.

We used the individual test results to identify the self-report discrepancies for the three Uganda surveys combined, allowing us to review four categories in more detail: the total population, PSRP, self-report discrepancies, and PSRP concluded to be negative. We examined demographics and ARV information, including sex, age, ever having taken ARVs, current ARVs, and ARVs detected, calculating the years since ARVs were first taken by comparing the year in which they were first taken to the year in which the participant was surveyed. We then plotted the age range and years on ARVs across the four categories.

Lastly, the individual test results for the three Uganda surveys were examined and used to categorize the PSRP with any or all non-positive results. We calculated the percent of those concluded to be negative as a proportion of all the PSRP and as a proportion of all of the self-report discrepancies, for each survey and overall. For the sensitivity analyses, jackknife weighting was used for the national surveys and in Ethiopia, while Taylor series was used for the smaller refugee survey, and 95% confidence intervals are included throughout. No weighting was used for the other analyses.

## 3. Results

### 3.1. Participants Self-Reported as Positive across 15 Surveys

From 2015 to 2021, there were participants in every survey who self-reported previous HIV-positive results while their RTs or follow-up testing produced non-positive results. As seen in Table 3, participants who self-reported HIV-positive results but were concluded to be HIV-negative were very rare, occurring in 0.02–0.41% of participants aged 15–59 years old and generally occurring 25 or fewer times per survey: as few as 4 times (Ethiopia in 2017) and at most 40 and 42 times (Eswatini and Uganda in 2016, respectively).

A sensitivity analysis (Table 4) compared the survey HIV prevalence (based on the survey’s final HIV status) to the conditional prevalence. The change in prevalence relative to the survey was particularly high in the refugee survey (6.79%), with a small sample size and low prevalence. The change was otherwise highest in Côte d’Ivoire (2.36%) and Uganda 2016 and 2020 (2.26% and 2.18%, respectively) and lowest in Malawi (0.37%).

**Figure 4:**
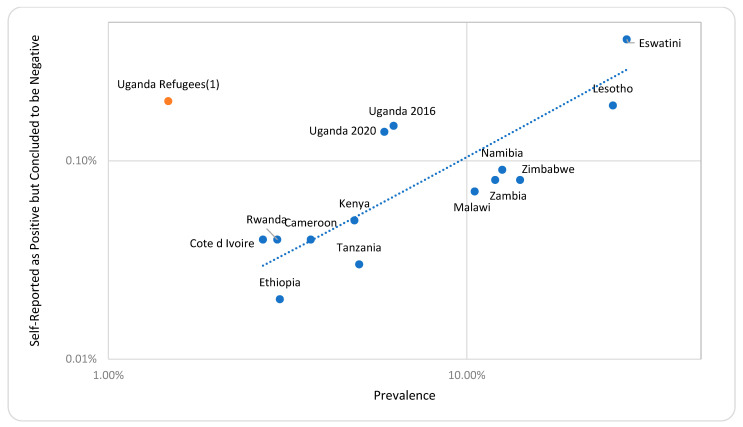
Frequency of population-based HIV impact assessment participants self-reporting HIV-positive results with a final survey status of HIV-negative among the participants versus country survey prevalence, with trendline; points are on a log–log scale for visibility. (1) Uganda Refugees survey is an outlier and not included in the trendline.

Comparing the PSRP concluded to be negative (among all the participants) and the survey prevalence, apart from the Uganda refugee survey, which had a small sample size, the percentage who were PSRP concluded to be negative trended higher in high-prevalence countries (Figure 4).

Our second sensitivity analysis (Table 5) showed that even if all the PSRP concluded to be negative had been HIV-positive with VLS, the VLS prevalence increase would have been as low as 0.12 percentage points (Malawi) and less than a percentage point in all but two surveys. In Côte d’Ivoire, with the lowest VLS, the VLS prevalence would have been 1.40 percentage points (3.57%) higher, and in the low-sample-size and low-HIV-prevalence refugee survey, VLS would have been 1.67 percentage points (2.26%) higher.

### 3.2. Participants Self-Reported as Positive and Self-Report Discrepancies in Uganda

**Figure 5:**
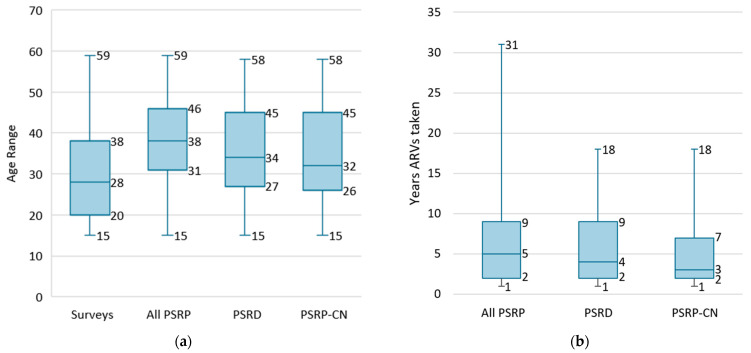
(**a**) Age range and (**b**) years since ARVs were first taken (only asked of PSRP) (for simplicity, only the year is used) among population-based HIV impact assessment participants who self-reported HIV-positive results (PSRP) aged 15–59 years old. Unweighted. Blue line: median; blue box: first quartile (Q1) through third quartile (Q3); whiskers: minimum and maximum. Across three Ugandan surveys from 2016 to 2021. PSRD: PSRP with discrepant (non-positive) test results; PSRP-CN: PSRP concluded to be negative.

When examining the demographics for the three Ugandan surveys (Table 6), males accounted for 42% of the participants overall, 42.1% of the self-report discrepancies, and 42.5% of those PSRP concluded to be HIV-negative. However, they were only 29.5% of the PSRP overall. Additionally (Figure 5a), we found that median age of the PSRP varied slightly compared to the survey population as a whole, but the first quartile to the third quartile overlapped; the median age among those with self-report discrepancies and among those PSRP concluded to be negative varied similarly compared to the PSRP. Self-report discrepancies, though rare, were found throughout the age range from 15 to nearly 59 years old.

Among the PSRP (Table 6), 91.6% reported ever having taken ARVs, but among those with discrepancies, only 57.9% did, and among those concluded to be negative, only 47.5% did. Similarly, among the PSRP who reported ever having taken ARVs, 97.6% reported taking them currently, but among those with discrepancies and those concluded to be negative, only 88.7% and 81.6%, respectively, reported current ARV use. Further, those with discrepancies (21.5%) and those concluded to be negative (28.8%) were far more likely not to answer the question about ever having taken ARVs than the PSRP (3.0%), though the follow-up question about whether they were currently taking ARVs (asked only of those who replied yes to ever having taken ARVs) was always answered. The detection of ARVs to corroborate self-reported ARV status gave similar results, with ARVs being detected in 84.0% of the PSRP but only 52.3% of those with discrepancies and 26.3% of those concluded to be negative.

Among the PSRP who reported when they first started taking ARVs, the median years for which ARVs had been taken varied slightly across groups (Figure 5b), though there was significant overlap. Neither those with discrepancies nor those concluded to be negative reported taking ARVs for more than 18 years, though the PSRP reported usage as long as 31 years.

Less than 5% of the PSRP in the Uganda 2016 and 2020 national surveys had any discrepant (non-positive) test results, though 21.6% did in the 2021 refugee survey; a total of seven (87.5%) of these eight individuals with discrepancies in 2021 had negative PCR results (Table 7). Most of those with discrepancies had entirely non-positive tests (74 (69.2%) out of 107, across all surveys). Among the 17 Uganda 2020 PSRP with WB results, 14 (82.4%) were negative. One PSRP who was negative on WB was positive on PCR; conversely, two PSRP who were positive on WB were negative on PCR. Among those with any discrepant results, most were concluded to be negative (84.0% for Uganda 2016, 67.3% for Uganda 2020, 62.5% for the refugee survey, and 74.8% overall).

## 4. Discussion

Despite the high-quality processes and sensitive and specific tests employed in these PHIA surveys, a small number of PSRP were concluded to be negative (less than 1.5% of the PSRP across 15 surveys); this was concerning but not numerous. These cases seem to occur more frequently among the population in high-prevalence countries, though a previous, more formal analysis found no strong correlation of these cases among PSRP with HIV prevalence [7]. There are several explanations for the discrepancies which must be considered.

This analysis encompassed a wider geographical scope and larger sample size compared to previous studies [1,2,3,4,5,6,7], providing a more comprehensive understanding of this understudied phenomenon. It examined similar data but focused on different details compared to a recent brief report also based on PHIA surveys [7].

Even if all the PSRP concluded to be negative had been positive, or if all the PSRP were considered to be positive without additional testing, their final statuses would not have significantly affected survey outcomes such as the prevalence of HIV or the prevalence of VLS. These changes are well within the 95% confidence intervals.

However, these results raise questions about how to handle these rare cases. Are they the result of incorrect tests or incorrect self-report statuses? Let us return to the five scenarios outlined above that would result in self-report discrepancies.

We saw that 75% of the participants with discrepancies in Uganda were concluded to be HIV-negative, leaving 25% who were concluded to be HIV-positive. Given the discrepancies among the recorded test results, the negative results for those concluded to be positive could have been the result of misreading or recording errors in the test results (scenario 1) or the failure of some tests to detect HIV (scenario 5). Returning to the 75% concluded to be negative, we can rule out scenario 1, misreading or recording errors in the test results, because of the very low likelihood of repeated recording errors, and the use of Geenius testing, which is neither transcribed nor read visually.

Now, we are left with scenarios 2, 3, 4, and 5 for the PSRP concluded to be negative. To distinguish these scenarios, we can review ARV detection.

Among the PSRP concluded to be negative in Uganda, 74% did not have detectable ARVs. Scenarios 2 and 4, an incorrect self-report status due to recording errors or misstatements, seem likely and have been seen anecdotally during PHIA surveys. We have described changes that were made to the questions that were asked in previous tests in Uganda—survey designers re-evaluate the questions to reduce the likelihood of incorrect answers [26]—which suggests that one set of questions might have produced incorrect answers in a few cases. Given the diverse nationalities of the participants in the Uganda Refugees 2021 survey, language challenges may have played a part for this survey, more so than in other surveys, and may have been another source of scenarios 2 and 4. However, among the 26% of PSRP concluded to be negative who were on ARVs, scenarios 2 and 4 can be eliminated because neither would explain access to ARVs.

Turning back to the PSRP concluded to be negative without detectable ARVs, scenarios 5 and 3, a previous diagnosis or misdiagnosis with lapsed ARV compliance, are possible, but the high prevalence of ARV usage among the PSRP suggests that ARV lapses are rare. Further, scenario 5, a true HIV diagnosis with lapsed ARV compliance but HIV remaining undetectable—post-treatment control—seems unlikely; this is less rare than those extremely few people who are no longer infected but still very rare [27,28].

For PSRP concluded to be negative with detectable ARVs, scenario 3 seems the most likely explanation, as a negative conclusion for the PSRP was generally supported by negative TNA PCR tests, and it is generally thought that laboratory tests such as these are accurate except in very rare cases [29]. However, given the one case in Uganda where their TNA PCR testing was negative but WB was positive, it is possible that in other PHIA surveys without WB testing, a very small number of the PSRP concluded to be negative might have fallen into scenario 5, participants who were living with HIV but whose infection had become hard to detect, a known outcome for those on ARVs [30,31,32]. (Notably, we did not find that those concluded to be negative had been on ARVs longer than the rest of the PSRP.)

Turning to the ethics of not causing harm to the participants, how should we handle each of these cases? During the initial household visit, the survey staff did not have sufficient evidence to distinguish which scenarios were more likely.

If there was misreading of the test results or the test failed to detect HIV in the household (scenarios 1 and 5), this could have caused these participants to doubt their HIV status or might have affected their ARV adherence. While this was mitigated by the counseling messages, prompt follow-up after discrepancy resolution for those concluded to be positive is very important.

Scenarios 2 and 4 caused some challenges for the survey but required minimal or no harm to the participant, as the participant did not actually believe they were living with HIV, and all their tests would have been negative. However, as mentioned, there would have been a household revisit to return their negative PCR results due to the possibility of scenario 3, particularly if their ARV detection results were not available yet. This revisit had the potential to cause confusion for scenario 2 and 4 participants and therefore had to be handled with care.

The most ethically concerning scenario was number 3, where the participant reported previous positive tests but those tests were incorrect, indicating a potential misdiagnosis and unnecessary ARV use and raising the question of how the survey could best communicate this finding to the participant and the ministry of health. Even with high sensitivity and specificity and high-quality processes, HIV misdiagnoses do occur [30]. National HIV testing algorithms evolve over time, as seen between the Uganda surveys, to minimize false positives and false negatives, giving insight into the complexities involved in testing and in designing algorithms. The 2015 WHO recommendations, for instance, indicated that the “tiebreaker” approach used in earlier algorithms increased the likelihood of misdiagnoses [25]. This misdiagnosis scenario could affect over 70,000 people across the first 11 PHIA countries alone [33], a significant ethical concern. Given the serious consequences [34,35,36] of remaining on unneeded ARV treatment, those who are not living with HIV (scenario 3) should not take ARVs (unless indicated for pre- or post-exposure prophylaxis). However, the risk of false negative results makes it challenging to review past diagnoses to distinguish these PSRP from scenario 5 (who must continue on ARVs to maintain their VLS), identify those with misdiagnoses, and correct their treatment. Therefore, during the household revisits to the PSRP concluded to be negative, the survey teams worked closely with each ministry of health to ensure that any cessation of ARV treatment be undertaken with close monitoring for viral resurgence.

There is an additional scenario which participants may wrongly believe is possible: that a participant formerly living with HIV is no longer infected. Survey staff know this is extremely unlikely, has been documented in very few cases worldwide, and generally has involved specific interventions [27,37,38,39,40]; ethics suggests that they ensure participants do not assume this scenario.

Self-reporting of previous HIV-positive tests has a very high correlation with people living with HIV [1,2,4,41,42,43,44], which suggests it might not be necessary to test these participants during a survey and thus avoid the risk of such discrepancies. Further, we found that the primary survey outcomes would not have changed significantly if these participants were assumed to be positive without testing.

However, there are several reasons to perform HIV testing in the household. One significant reason is to reduce stigma; if PSRP are not tested, this may be noticed by others in the household or the neighborhood, even with significant attempts to achieve privacy for the interview and testing. Another reason is the risk that PSRP are actually negative (scenarios 3 and 4) and will therefore have no detectable VL, falsely (though minimally) inflating the prevalence of VLS. While there are non-testing methods for confirming the results for PSRP, such as requesting to see ARV bottles or treatment identity cards [45], these may be invasive and reduce confidentiality. Additionally, since blood is collected from all participants, whether to test for HIV or to determine the prevalence of VLS, it is not a significant burden on the participant to have RTs run in their household, and by simplifying the logic for who are tested, the likelihood of missed tests is reduced.

Given these reasons for testing PSRP, surveys must maintain ethical standards of treatment for participants that recognize the possibility of finding such cases and are prepared to manage them, including whether and how to inform the participants and whether to include this risk in the consent forms. The survey protocols should reflect national guidelines, but guidelines for dealing with participants (or patients) with self-report discrepancies, particularly those for whom all available HIV tests are negative, are not always available.

At the same time, surveys are not the only situations in which people living with HIV who know their status might be retested, whether individually or in larger groups [5,46]. Given the potential for negative results, guidelines are needed more generally, optimally at the national or global level, not only on how to manage such discrepancies but on how to track potential misdiagnoses. Such guidelines might also identify those at greatest risk of misdiagnosis and how to proactively address this.

## 5. Conclusions

As we saw in the evolution of the self-report questions for Uganda, survey designers re-evaluate questions to reduce the likelihood of incorrect answers, but incorrect answers can still be recorded. At the same time, HIV diagnostic testing has been updated over time but is designed for patients who are treatment-naïve. While it is known that RTs may result in false negatives for those on treatment, it is generally thought that laboratory tests such as TNA PCR are accurate except in very rare cases. However, for some of the PHIA participants, even their PCR results occasionally contradicted the other evidence, such as WB results. Household surveys should acknowledge such complexities if they are to ethically report their results to participants without causing harm; even in a subnational survey such as Uganda Refugees 2021 (with just over 2500 participants), such cases may be found. Given the serious consequences of remaining on unneeded ARV treatment, ethics suggests that guidelines could be written and more research could be undertaken to determine how to confirm a suspected HIV misdiagnosis, and for people who are confirmed to be living with HIV who sometimes test negative, whether it makes sense to continue a treatment plan designed for those with more concordant results.

## Figures and Tables

**Table 1:** Questions used in Uganda population-based HIV impact assessment surveys to determine results of previous HIV tests.

Question	Uganda 2016	Uganda 2020 and Uganda Refugees 2021
What month and year was your last HIV test? What was the result of that HIV test?	included	not included
When was your last HIV test? Please give the month and year if you can. What was the result of your last HIV test?	not included	included
Has a health care provider ever told you that you have HIV?	not included	included
Did you test positive for HIV before your pregnancy with [child’s name]?	included ^1^	included ^1^
What was the result of your last HIV test during your last pregnancy with [child’s name]?	included ^1^	included ^1^
Did you test for HIV during labor? What was the result of that test?	included ^1,2^	not included
What was the result of the HIV test that you received after the delivery of your last pregnancy with [child’s name]?	not included	included ^1^

^1^ Included for participants who had experienced a pregnancy. ^2^ Included but redacted from the public dataset.

**Table 2:** Possibilities for self-reporting of previous HIV-positive results with any discrepant HIV tests; what is true?

	Self-Report Is True	Self-Report Is False
Test is correct	The specimen correctly tested positive but was misread or recorded incorrectly during the data collection. The participant correctly reported no previous positive tests but recorded this incorrectly during the data collection.	3. The participant reported previous positive results but had been misdiagnosed. 4. The participant falsely reported previous positive results for reasons unknown.
Test is incorrect	5. Testing failed to detect HIV.	Not applicable

**Table 3:** Participants of population-based HIV impact assessments who self-reported HIV-positive results, aged 15–59 years old, across fifteen surveys from 2015 to 2021.

Country	Survey	Year Started	Participants	Tested HIV-Positive	Viral Load Suppressed ^1^	PSRP	PSRP-CN, % of Participants	PSRP-CN, % of PSRP
Cameroon	CAMPHIA	2017	25,279	944	406	432	10/25,279 (0.04%)	10/432 (2.31%)
Côte d’Ivoire	CIPHIA	2017	17,319	417	181	161	7/17,319 (0.04%)	7/161 (4.35%)
Eswatini	SHIMS2	2016	9744	2839	2096	2486	40/9744 (0.41%)	40/2486 (1.61%)
Ethiopia ^2^	EPHIA	2017	18,563	593	417	453	4/18,563 (0.02%)	4/453 (0.88%)
Kenya	KENPHIA	2018	27,139	1482	1068	1097	14/27,139 (0.05%)	14/1097 (1.28%)
Lesotho	LEPHIA	2016	11,867	3239	2238	2582	23/11,867 (0.19%)	23/2582 (0.89%)
Malawi	MPHIA	2015	16,869	2176	1482	1606	11/16,869 (0.07%)	11/1606 (0.68%)
Namibia	NAMPHIA	2017	16,292	2353	1818	1921	14/16,292 (0.09%)	14/1921 (0.73%)
Rwanda	RPHIA	2018	29,476	888	680	661	13/29,476 (0.04%)	13/661 (1.97%)
Tanzania	THIS	2016	29,287	1771	924	1055	10/29,287 (0.03%)	10/1055 (0.95%)
Uganda 2016	UPHIA	2016	28,593	1735	1051	1201	42/28,593 (0.15%)	42/1201 (3.50%)
Uganda 2020	UPHIA	2020	23,148	1388	1055	1081	33/23,148 (0.14%)	33/1081 (3.05%)
Uganda Refugees ^3^	RUPHIA	2021	2509	44	34	37	5/2509 (0.20%)	5/37 (13.51%)
Zambia	ZAMPHIA	2016	19,592	2527	1508	1738	15/19,592 (0.08%)	15/1738 (0.86%)
Zimbabwe	ZIMPHIA	2015	19,970	3293	2057	2515	15/19,970 (0.08%)	15/2515 (0.60%)
Overall	-	-	295,647	25,689	17,015	19,026	256/295,647 (0.09%)	256/19,026 (1.35%)

^1^ Viral load suppressed, <1000 copies per milliliter, among those concluded to be HIV-positive. ^2^ Ethiopia is urban-only. ^3^ Uganda Refugees is a subnational population. PSRP: participants who self-reported previous positive results; PSRP-CN: PSRP concluded to be HIV-negative.

**Table 4:** Sensitivity analyses for HIV prevalence among people aged 15–59 years old, conditionally considering those who self-reported HIV-positive results with a final survey status of HIV-negative to be positive, across fifteen population-based HIV impact assessment surveys from 2015 to 2021.

Survey	Survey Prevalence ^1^ (95% Confidence Interval)	Conditional Prevalence ^2^ (95% Confidence Interval)	Percentage Point Increase (Absolute Change)	Increase as a Percent of Prevalence (Relative Change)
Cameroon	3.67% (3.32–4.02%)	3.71% (3.36–4.06%)	0.04%	1.06%
Côte d’Ivoire	2.70% (2.37–3.03%)	2.77% (2.42–3.11%)	0.07%	2.36%
Eswatini	27.92% (26.54–29.30%)	28.35% (26.96–29.73%)	0.43%	1.53%
Ethiopia ^3^	3.01% (2.60–3.42%)	3.03% (2.62–3.45%)	0.02%	0.67%
Kenya	4.86% (4.47–5.24%)	4.89% (4.50–5.28%)	0.03%	0.68%
Lesotho	25.56% (24.68–26.44%)	25.74% (24.86–26.62%)	0.18%	0.72%
Malawi	10.52% (9.88–11.16%)	10.56% (9.92–11.20%)	0.04%	0.37%
Namibia	12.56% (11.68–13.45%)	12.65% (11.76–13.53%)	0.09%	0.68%
Rwanda	2.96% (2.62–3.31%)	3.02% (2.67–3.36%)	0.06%	1.72%
Tanzania	5.01% (4.66–5.37%)	5.03% (4.68–5.39%)	0.02%	0.41%
Uganda 2016	6.25% (5.82–6.67%)	6.39% (5.96–6.82%)	0.14%	2.26%
Uganda 2020	5.89% (5.40–6.39%)	6.02% (5.53–6.52%)	0.13%	2.18%
Uganda Refugees ^4^	1.47% (0.83–2.11%)	1.57% (0.93–2.21%)	0.10%	6.79%
Zambia	12.00% (11.34–12.66%)	12.08% (11.42–12.74%)	0.08%	0.68%
Zimbabwe	14.08% (13.42–14.75%)	14.15% (13.49–14.82%)	0.07%	0.50%
Overall ^5^	9.23% (N/A)	9.33% (N/A)	0.10%	1.08%

^1^ Survey prevalence is based on participants with a final survey status of HIV-positive. ^2^ Conditional prevalence adds participants who self-reported HIV-positive results with a final survey status of HIV-negative to those with a final survey status of HIV-positive. ^3^ Ethiopia is urban-only. ^4^ Uganda Refugees is a subnational population. ^5^ Overall shows average of the prevalence columns; difference values are calculated for that row. N/A: not available.

**Table 5:** Sensitivity analysis of population HIV viral load suppression, <1000 copies per milliliter, among all HIV-positive people aged 15–59 years old, conditionally considering those who self-reported HIV-positive results with a final survey status of HIV-negative as positive with viral load suppression; across fifteen population-based HIV impact assessment surveys from 2015 to 2021.

Survey	Survey Population VLS ^1^ (95% Confidence Interval)	Conditional Population VLS ^2^ (95% Confidence Interval)	Percentage Point Increase (Absolute Change)	Increase as a Percent of Population VLS (Relative Change)
Cameroon	43.99% (39.97–48.02%)	44.58% (40.65–48.51%)	0.59%	1.34%
Côte d’Ivoire	39.27% (32.07–46.48%)	40.68% (33.62–47.74%)	1.41%	3.57%
Eswatini	72.37% (70.43–74.30%)	72.78% (70.87–74.69%)	0.41%	0.57%
Ethiopia ^3^	70.26% (65.43–75.09%)	70.45% (65.64–75.27%)	0.19%	0.28%
Kenya	70.81% (67.92–73.69%)	71.00% (68.12–73.88%)	0.19%	0.28%
Lesotho	67.57% (65.68–69.45%)	67.80% (65.94–69.65%)	0.23%	0.34%
Malawi	67.74% (65.39–70.08%)	67.85% (65.52–70.19%)	0.11%	0.17%
Namibia	76.89% (74.26–79.52%)	77.04% (74.42–79.67%)	0.15%	0.20%
Rwanda	75.66% (71.55–79.77%)	76.07% (72.03–80.12%)	0.41%	0.54%
Tanzania	51.91% (48.51–55.30%)	52.10% (48.73–55.48%)	0.19%	0.38%
Uganda 2016	59.31% (56.39–62.24%)	60.21% (57.31–63.12%)	0.90%	1.52%
Uganda 2020	74.52% (71.63–77.42%)	75.07% (72.24–77.90%)	0.55%	0.73%
Uganda Refugees ^3^	73.82% (60.21–87.43%)	75.48% (62.67–88.30%)	1.66%	2.26%
Zambia	57.80% (55.28–60.31%)	58.08% (55.59–60.57%)	0.28%	0.49%
Zimbabwe	58.96% (56.75–61.16%)	59.16% (56.97–61.35%)	0.20%	0.34%
Overall ^4^	64.06% (N/A)	64.56% (N/A)	0.50%	0.78%

^1^ Survey VLS is based on participants with a final survey status of HIV-positive. ^2^ Conditional VLS adds participants who self-reported HIV-positive results with a final survey status of HIV-negative to the those with a final survey status of HIV-positive. ^3^ Ethiopia is urban-only. Uganda Refugees is a subnational population. ^4^ Overall shows average of population VLS columns; difference values are calculated for that row. VLS: viral load suppression. N/A: not available.

**Table 6:** Demographics and anti-retroviral information among participants who self-reported HIV-positive results, those with discrepant survey test results, and those concluded to be negative, aged 15–59 years old across three population-based HIV impact assessment surveys in Uganda from 2016 to 2021.

		Surveys Overall ^1^	PSRP	Discrepancy	Concluded to Be Negative
Total participants		54,250	2319	107	80
Male		22,764 (42.0%)	685 (29.5%)	45 (42.1%)	34 (42.5%)
Female		31,486 (58.0%)	1634 (70.5%)	62 (57.9%)	46 (57.5%)
ARVs taken ever (only asked of PSRP)	Yes	N/A	2125 (91.6%)	62 (57.9%)	38 (47.5%)
	No	N/A	125 (5.4%)	22 (20.6%)	19 (23.8%)
	No answer ^2^	N/A	69 (3.0%)	23 (21.5%)	23 (28.8%)
ARVs taken currently (if ARVs taken ever, Yes)	Yes	N/A	2075 (97.6%)	55 (88.7%)	31 (81.6%)
	No	N/A	50 (2.4%)	7 (11.3%)	7 (18.4%)
	No answer ^2^	N/A	0 (0.0%)	0 (0.0%)	0 (0.0%)
ARVs detected ^3^	Tested	N/A	2274	65	38
	Detected	N/A	1910 (84.0%)	34 (52.3%)	10 (26.3%)
	Not detected	N/A	364 (16.0%)	31 (47.7%)	28 (73.7%)

^1^ Those with a final survey HIV status. ^2^ “No answer” includes “Don’t Know”, “Refused”, and missing values. ^3^ ARV testing was carried out for all participants concluded to be positive; for the two recent surveys, all PSRP were tested, even those concluded to be negative. Percents are among rows within the same section; ARV: antiretroviral; Q1: first quartile; Q3: third quartile; Discrepancy: self-reported previous positive tests with discrepant (non-positive) survey test results; PSRP: participants who self-reported previous positive tests. N/A: not applicable.

**Table 7:** Frequency of discrepant (non-positive) results among participants who self-reported positive results aged 15–59 years old across three population-based HIV impact assessment surveys in Uganda from 2016 to 2021.

Survey	PSRP	Participants with Non-Positive Results for Each Test n/N (%)							PSRP-CN among PSRP	PSRP-CN among PSRD
		Household RTs	Quality Assurance RTs ^1^	Geenius ^2^	TNA PCR ^3^	Western Blot ^4^	Any Test (PSRD)	All Tests ^5^		
Uganda 2016	1201	43/1201 (3.6%)	41/1183 (3.5%)	N/A	40/43 (93.0%)	N/A	50/1201 (4.2%)	36/1201 (3.0%)	42/1201 (3.5%)	42/50 (84.0%)
Uganda 2020	1081	46/1080 (4.3%)	15/119 (12.6%)	39/1081 (3.6%)	36/44 (81.8%)	14/17 (82.4%)	49/1081 (4.5%)	32/1081 (3.0%)	33/1081 (3.1%)	33/49 (67.3%)
Uganda Refugees ^6^	37	7/37 (18.9%)	6/13 (46.2%)	8/37 (21.6%)	7/8 (87.5%)	N/A	8/37 (21.6%)	6/37 (16.2%)	5/37 (13.5%)	5/8 (62.5%)
Total	2319	96/2318 (4.1%)	62/1315 (4.7%)	47/1118 (4.2%)	83/95 (87.4%)	14/17 (82.4%)	107/2319 (4.6%)	74/2319 (3.2%)	80/2319 (3.4%)	80/107 (74.8%)

^1^ Quality assurance was conducted for the majority of participants in Uganda 2016 but for only a select subset in the other surveys. ^2^ Geenius was not used for Uganda 2016. ^3^ TNA PCR was only run for PSRP when at least one test was non-positive. ^4^ Western Blot was only run for PSRP when at least one test was non-positive and only for Uganda 2020. ^5^ “All” among available results, i.e., non-positive results or tests not run for each test type. n: number non-positive; N: number tested; N/A: not available; RT: rapid test; PSRD: participants who self-reported previous positive results with discrepant (non-positive) survey test results; PSRP: participants who self-reported previous positive results; PSRP-CN: PSRP concluded to be negative; TNA PCR: total nucleic acid polymerase chain reaction. ^6^ Uganda Refugees is a subnational population.

## Data Availability

The public data presented in this study are openly available at https://phia-data.icap.columbia.edu/ (accessed on 15 September 2024). The process data are available on request from the corresponding author due to the small number of participants and privacy concerns.
